# Research of Koch’s Bacillus by Homogenization of the Sputum without Centrifugation

**Published:** 1919

**Authors:** 


**Research of Koch's Bacillus by Homogenization of the Sputum without Centrifugation.** By G. LAROCHE and VIRMEAUX. *Société de Biologie*, November 23, 1918.

The authors recommend the following method for the research of Koch's bacillus :

1. The sputum is homogenized by a sodium lixivium according to Bezançon and Philibert's method, i. e., 10 volumes of water for one of sputum. The sputum and half the volume of water are placed in a porcelain capsule, and sodium lixivium is added in the proportion of one drop per cc. of sputum. The remaining water is then slowly poured and warmed for about 10 minutes.

2. Chemically pure, Sodium chlorid is added in the proportion of 0 gr. 25 per cc., and mixed until the dissolution of the chlorid is complete.

3. The liquid is poured into a tube and, once it is cold, a few drops of an equal mixture of ether and ligroin are added. The tube is then violently shaken.

4. The bacilli are left to collect for 6 hours.

5. The bacilli are looked for in the thin grey layer formed under the layer of ligroin and collected by means of a pipette. They are then plated, dried and fixed by alcohol.

6. The plate is placed in distilled water and balanced until the precipitated chlorid dissolves.

7. The Koch bacilli are stained by Bezancon and Philibert's method.

This method seems to be indicated for the laboratories which do not possess any turbines.

